# Different Risk for Hypertension, Diabetes, Dyslipidemia, and Hyperuricemia According to Level of Body Mass Index in Japanese and American Subjects

**DOI:** 10.3390/nu10081011

**Published:** 2018-08-03

**Authors:** Masanari Kuwabara, Remi Kuwabara, Koichiro Niwa, Ichiro Hisatome, Gerard Smits, Carlos A. Roncal-Jimenez, Paul S. MacLean, Joseph M. Yracheta, Minoru Ohno, Miguel A. Lanaspa, Richard J. Johnson, Diana I. Jalal

**Affiliations:** 1 Department of Cardiology, Toranomon Hospital, Tokyo 105-8470, Japan; minotky@gmail.com; 2 Division of Renal Diseases and Hypertension, School of Medicine, University of Colorado Denver, Aurora, CO 80045, USA; smits.gerard.j@gmail.com (G.S.); Carlos.Roncal@ucdenver.edu (C.A.R.-J.); Miguel.LanaspaGarcia@ucdenver.edu (M.A.L.); Richard.Johnson@ucdenver.edu (R.J.J.); 3 Cardiovascular Center, St. Luke’s International Hospital, Tokyo 104-8560, Japan; kniwa@aol.com; 4 Department of Pediatrics, Nihon University School of Medicine, Tokyo 173-8610, Japan; 5 Division of Regenerative Medicine and Therapeutics, Department of Regenerative Medicine and Genomic Function, Institute of Regenerative Medicine and Biofunction, Tottori University Graduate School of Medical Science, Yonago, Tottori 683-8503, Japan; hisatome@med.tottori-u.ac.jp; 6 Division of Endocrinology, Metabolism and Diabetes, School of Medicine, University of Colorado Denver, Aurora, CO 80045, USA; Paul.MacLean@ucdenver.edu; 7 Department of Pharmaceutics, University of Washington, Seattle, WA 98195, USA; jmy5@uw.edu; 8 Department of Medicine, University of Iowa, Iowa, IA 52242, USA; diana-jalal@uiowa.edu

**Keywords:** body mass index, hypertension, epidemiology, risk factor

## Abstract

Obesity is a risk factor for hypertension, diabetes mellitus (DM), dyslipidemia, and hyperuricemia. Here, we evaluated whether the same body mass index (BMI) for the U.S. population conferred similar metabolic risk in Japan. This was a cross-sectional analysis involving 90,047 Japanese adults (18–85 years) from St. Luke’s International Hospital, Tokyo, Japan and 14,734 adults from National Health and Nutrition Examination Survey (NHANES) collected in the U.S. We compared the prevalence of hypertension, DM, dyslipidemia, and hyperuricemia according to BMI in Japan and the U.S. The prevalence of hypertension, DM, and dyslipidemia were significantly higher in the U.S. than Japan, whereas the prevalence of hyperuricemia did not differ between countries. Higher BMI was an independent risk factor for hypertension, DM, dyslipidemia, and hyperuricemia both in Japan and in the U.S. after adjusting for age, sex, smoking and drinking habits, chronic kidney disease, and other cardiovascular risk factors. The BMI cut-off above which the prevalence of these cardio-metabolic risk factors increased was significantly higher in the U.S. than in Japan (27 vs. 23 kg/m^2^ for hypertension, 29 vs. 23 kg/m^2^ for DM, 26 vs. 22 kg/m^2^ for dyslipidemia, and 27 vs. 23 kg/m^2^ for hyperuricemia). Higher BMI is associated with an increased prevalence of hypertension, DM, dyslipidemia, and hyperuricemia both in Japan and U.S. The BMI cut-off above which the prevalence of cardio-metabolic risk factors increases is significantly lower in Japan than the U.S., suggesting that the same definition of overweight/obesity may not be similarly applicable in both countries.

## 1. Introduction

The prevalence of obesity, a well-known risk factor for hypertension, diabetes mellitus (DM), and cardiovascular disease is increasing worldwide [1,2]. Globally, age-standardized mean body mass index (BMI) increased from 21.7 kg/m^2^ in 1975 to 24.2 kg/m^2^ in 2014 in men, and from 22.1 kg/m^2^ in 1975 to 24.4 kg/m^2^ in 2014 in women. The prevalence of obesity increased more than three times in men and more than twice in women in the last four decades [1]. This is worrisome as obesity is recognized as an important cardio-metabolic risk factor [3]. Generally, obesity is believed to be less common in Asian countries than in the United States (U.S.) [4], possibly as a consequence of a healthier diet [5]. Asian subjects with metabolic syndrome or DM show modest increases in waist circumference and weight, but this is reportedly less than that observed in the U.S. [6]. The World Health Organization (WHO) had previously identified that a BMI of 23 kg/m^2^ may represent an increased risk in Asian populations [7]. Considering the increase in global age-standardized BMI, we hypothesized that the prevalence of obesity has increased in Japan in the last decade. Furthermore, we hypothesized that the cardio-metabolic risk factors associated with obesity will correlate with increased BMI, similar to the U.S. population.

## 2. Materials and Methods

### 2.1. Study Population

This is a retrospective cross-sectional study. We analyzed the database from The Center for Preventive Medicine at St. Luke’s International Hospital in Japan between 2004 and 2010, and the National Health and Nutrition Examination Survey (NHANES) database in the U.S. between 2001 and 2006. Details of the database at The Center for Preventive Medicine in Japan have been published previously [8,9,10,11,12,13]. Briefly, we included 90,047 adults (18–85 years) who underwent annual regular health check-up at the center. When the study subjects had exams more than once between 2004 and 2010, we adopted only the first results to avoid double counts. The NHANES is an ongoing program of studies designed to assess the health and nutritional status of adults in the U.S. [14]. This study sample was composed of data pooled from 3 waves of NHANES data that were collected during 2001–2006, which had already used an appropriate weight, based on the variables selected. Data were retained for analysis of 14,734 adults who had complete data for this study.

### 2.2. Definition of the Cardio-Metabolic Risk Factors

Hypertension was defined as taking antihypertensive medications or a systolic blood pressure (BP) of ≥40 mmHg and/or a diastolic BP of ≥90 mmHg according to the Japanese Society of Hypertension guidelines (JSH 2014) [15]. In Japan, BP was recorded using an automatic brachial sphygmomanometer (OMRON Corporation, Kyoto, Japan). BPs of the individual were measured twice after taking the sitting position and remaining quiet for longer than 5 min, with the feet on the ground and the back supported. In NHANES, BP was recorded using a mercury manometer. After resting quietly in a sitting position for 5 min and determination of the maximum inflation level, up to 4 consecutive BP readings were obtained. The mean values of systolic and diastolic BP of each individual were calculated from the recorded measurements. DM was defined as taking glucose lowering therapies or a glycated hemoglobin (HbA_1c_) concentration of ≥6.5% (as per National Glycohemoglobin Standardization Program) according to International Expert Committee [16]. Dyslipidemia was defined as taking lipid lowering medications or a low-density lipoprotein cholesterol level of ≥140 mg/dL, a high-density lipoprotein cholesterol level of <40 mg/dL, or a triglyceride level of ≥150 mg/dL according to Japan Atherosclerosis Society guidelines [17]. Hyperuricemia was defined as taking uric acid lowering medications or a serum uric acid concentration of >7.0 mg/dL according to Japanese Guideline for the Management of Hyperuricemia and Gout: Second Edition [18].

### 2.3. Definition of Other Covariates

Chronic kidney disease was defined as the subjects whose estimated glomerular filtration rate (eGFR) < 60 mL/min/1.73 m^2^. We calculated eGFR using the Japanese eGFR equation as eGFR (mL/min/1.73 m^2^) = 194 × serum creatinine (−1.094) × Age (−0.287) × 0.739 (if female) for Japanese [19] and using Modification of Diet in Renal Disease (MDRD) equation for American subjects [20]. Smoking was defined as both former smokers and current smokers. Drinking habits was defined as both social drinkers and daily drinkers.

### 2.4. Statistical Analysis

**Figure 1:**
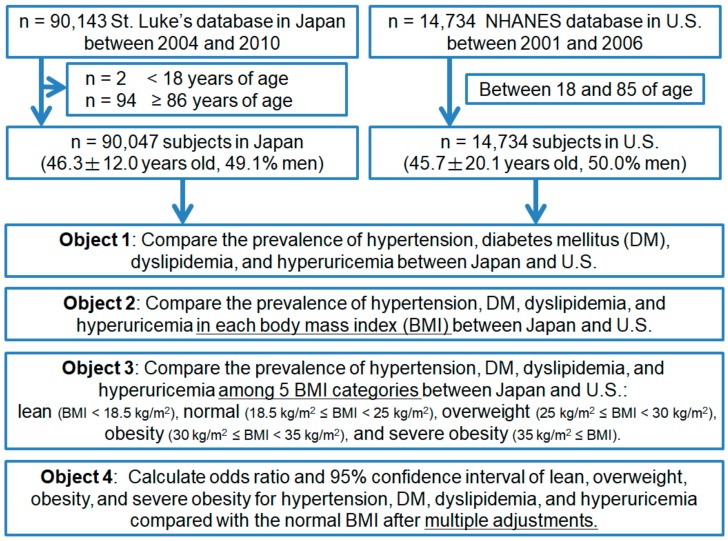
Flow diagram of study design.

We compared the prevalence of hypertension, DM, dyslipidemia, and hyperuricemia between the U.S. and Japan. We also compared the prevalence of hypertension, DM, dyslipidemia, and hyperuricemia according to BMI between the U.S. and Japan. BMI was categorized based on the WHO classification of adults according to BMI: BMI < 18.5 kg/m^2^ as lean, from 18.5 to 25 kg/m^2^ as normal, from 25 to 30 kg/m^2^ as overweight, 30 kg/m^2^≤ as obesity, 35 kg/m^2^≤ as severe obesity [21,22]. We also compared the continuous relationship of BMI with the various metabolic disorders. We evaluated the BMI cut-off point above which the prevalence of hypertension, DM, dyslipidemia, and hyperuricemia increased in both countries. We calculated odds ratios (ORs) and their 95% confidence intervals (CIs) for hypertension, DM, dyslipidemia, and hyperuricemia by BMI of 1 kg/m^2^ increased and each BMI classification after multiple adjustments with age, sex, smoking and drinking habits, chronic kidney disease, and other diseases (hypertension, DM, dyslipidemia, and hyperuricemia) (Figure 1).

Statistical significance was defined as *p* = 0.05, and all statistical analyses were two-sided. Data re expressed as mean ± standard derivation or as percent frequency, unless otherwise specified. Comparisons between two groups were performed with *t*-tests for normally distributed variables, and χ^2^ analyses for categorical data. Pairwise comparison of the prevalence of hypertension, DM, dyslipidemia, and hyperuricemia between Japan and U.S. in each BMI were performed by χ^2^ analyses. The risk factors for each disease were evaluated by multivariable logistic regression models with adjustments for age, sex, smoking and drinking habits, chronic kidney disease, and other cardio-metabolic risk factors (hypertension, DM, dyslipidemia, and hyperuricemia), and continuous value of BMI or five categories of BMI; lean (BMI < 18.5 kg/m^2^), normal (18.5 kg/m^2^ ≤ BMI < 25 kg/m^2^), overweight (25 kg/m^2^ ≤ BMI < 30 kg/m^2^), obesity (30 kg/m^2^ ≤ BMI < 35 kg/m^2^), and severe obesity (35 kg/m^2^ ≤ BMI) as WHO classification [21,22]. Age was defined as a continuous variable in years, sex defined as male and female. All statistical analyses were performed using the SPSS Statistics software (IBM SPSS Statistics version 22 for Windows; IBM, New York, NY, USA).

### 2.5. Ethical Considerations

We adhere to the principles of the Declaration of Helsinki. All data were collected and compiled in a protected computer database. Individual data were kept anonymous and there was no personality information identified. St. Luke’s International Hospital Ethics Committee approved the protocol for this study. We had consents from all the Japanese subjects by comprehensive agreement method in the hospital. During the informed consent process of NHANES, survey participants are assured that data collected will be used only for stated purposes and will not be disclosed or released to others without the consent of the individual or the establishment in accordance with Section 308 (d) of the Public Health Service Act (42 U.S.C. 242 m).

## 3. Results

### 3.1. Study Subjects Characteristics

**Figure 2:**
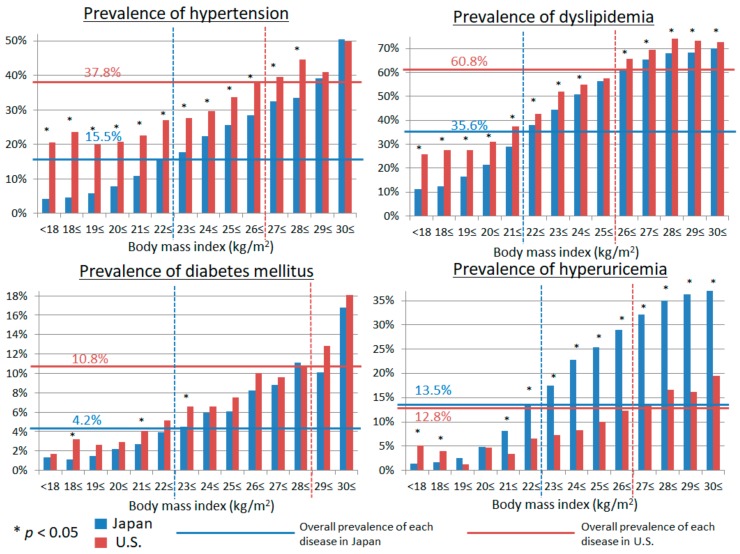
The prevalence of hypertension, diabetes mellitus, dyslipidemia, and hyperuricemia in each body mass index between Japan and the United States of America (U.S.). Solid blue lines showed mean prevalence of each disease in Japan and solid red lines showed the mean prevalence of each disease in the U.S. Dashed lines showed the proper cut-off points of body mass index for each disease, which shows higher than mean prevalence of each disease.

The characteristics of the 90,047 subjects in Japan and 14,734 subjects in the U.S. are shown in Table 1. The Japanese subjects were slightly older and had smaller height, lighter weight, lower BMI, and less smoking and drinking habits. The prevalence of hypertension, DM, and dyslipidemia was significantly higher in the U.S. population than in Japan. In contrast, the prevalence of hyperuricemia in the U.S. was slightly lower than in Japan (Figure 2, solid lines).

### 3.2. The Prevalence of Cardio-Metabolic Risk Factors in Relation to BMI in Japan and U.S.

**Figure 3:**
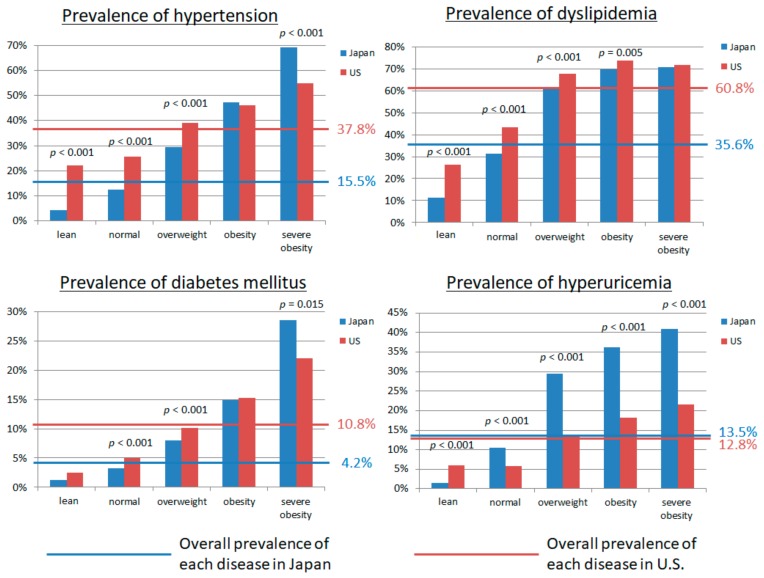
The prevalence of hypertension, diabetes mellitus, dyslipidemia, and hyperuricemia in each classification of body mass index (lean, normal, overweight, obesity, and severe obesity) between Japan and the United States of America (U.S.). *p* value < 0.05 shows significant difference of prevalence of each disease between Japan and the U.S. by χ^2^ analyses.

**Figure 4:**
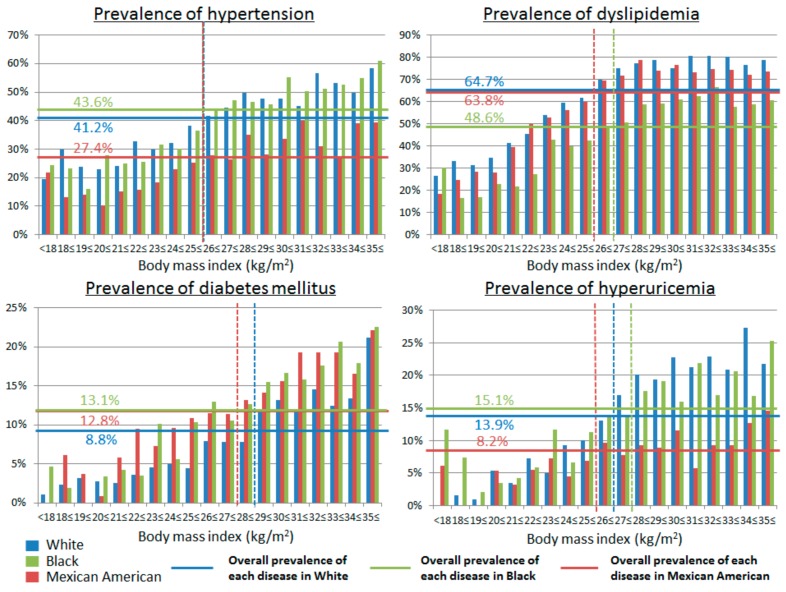
The prevalence of hypertension, diabetes mellitus, dyslipidemia, and hyperuricemia among White Americans, Black Americans, and Mexican Americans. Solid blue lines showed mean prevalence of each disease in White American, solid green lines showed mean prevalence of Black Americans, and solid red lines showed the mean prevalence in Mexican Americans. Dashed lines showed the proper cut-off points of body mass index for each disease, which shows higher than mean prevalence of each disease.

We evaluated the prevalence of hypertension, DM, dyslipidemia, and hyperuricemia in Japan and the U.S. for each BMI according to universally defined BMI categories (Figure 3). We also examined the prevalence of hypertension, DM, dyslipidemia, and hyperuricemia in Japan and the U.S. for potential BMI cut-offs (Figure 2). Lower BMI associated with a lower prevalence of hypertension, DM, dyslipidemia, and hyperuricemia both in Japan and the U.S. The BMI cut-off above which the prevalence of hypertension increased was higher in the U.S. than in Japan (27 vs*.* 23 kg/m^2^). We observed the same for DM (BMI 29 in the U.S. vs. 23 kg/m^2^ in Japan), dyslipidemia (26 vs. 22 kg/m^2^), and hyperuricemia (27 vs. 23 kg/m^2^). Of note, neither race nor ethnicity impacted the association between BMI and cardio-metabolic risk factors in the U.S. (Figure 4).

### 3.3. Multivariable Regression Analysis

**Figure 5:**
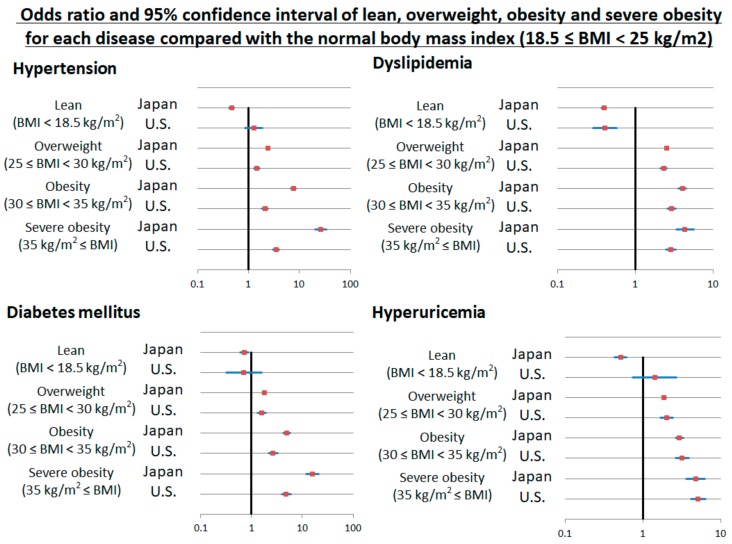
Odds ratio and 95% confidence interval of lean, overweight, obesity, and severe obesity for hypertension, diabetes mellitus, dyslipidemia, and hyperuricemia compared with the normal body mass index (18.5 ≤ BMI < 25 kg/m^2^).

After adjusting for age, sex, BMI, smoking and drinking habits, chronic kidney disease and the other cardio-metabolic risk factors, subjects in the U.S. had higher ORs for hypertension (OR: 1.443; 95% CI, 1.360–1.532; *p* < 0.001) and dyslipidemia (OR: 1.502; 95% CI, 1.428–1.581; *p* < 0.001), but lower ORs for DM (OR: 0.744; 95% CI, 0.680–0.813; *p* < 0.001) and hyperuricemia (OR: 0396; 95% CI, 0.368–0.426; *p* < 0.001) compared to Japanese subjects. Higher BMI was an independent risk factor for hypertension, DM, dyslipidemia, and hyperuricemia both in Japan and U.S. These data are shown in detail in Table 2. Each 1 kg/m^2^ increase in BMI in Japanese was associated with a higher odds of cardio-metabolic risk factors than in the U.S. As previously shown, lower (lean) BMI in the U.S. associated with a lower risk for only dyslipidemia (OR: 0.404, 95% CI, 0.286–0.570), but not for hypertension, DM, or hyperuricemia compared with normal BMI. In contrast, lean BMI in Japan associated with a lower risk of hypertension (OR: 0.463, 95% CI, 0.413–0.519), DM (OR: 0.727, 95% CI, 0.593–0.890), dyslipidemia (OR: 0.397, 95% CI, 0.369–0.426), and hyperuricemia (OR: 0.514; 95% CI, 0.428–0.618) than normal BMI. These data are shown in Figure 5.

Lean Japanese had lower risk for hypertension (OR: 0.463, 95% CI, 0.413–0.519), DM (OR: 0.727, 95% CI, 0.593–0.890), dyslipidemia (OR: 0.397, 95% CI, 0.369–0.426), and hyperuricemia (OR: 0.514; 95% CI, 0.428–0.618) than normal weight Japanese. Lean American had lower risk for only dyslipidemia (OR: 0.404, 95% CI, 0.286–0.570), but not risk for hypertension (*p* = 0.24), DM (*p* = 0.40), and hyperuricemia (*p* = 0.30) compared with normal American. Overweight, obesity, and severe obesity became significantly higher risk factors for hypertension (OR: 2.369, 7.603, 26.16 in Japan, 1.445, 2.091, 3.445 in the U.S., respectively), DM (OR: 1.803, 4.940, 15.73 in Japan, 1.580, 2.668, 4.829 in the U.S., respectively), dyslipidemia (OR: 2.546, 4.017, 4.254 in Japan, 2.313, 2.947, 2.846 in the U.S., respectively), and hyperuricemia (OR: 1.867, 2.929, 4.738 in Japan, 2.027, 3.196, 5.099 in the U.S., respectively) compared with normal both in Japan and U.S. Hypertension: Data adjusted with age, sex, smoking and drinking habits, chronic kidney disease, and other diseases (diabetes mellitus, dyslipidemia, and hyperuricemia). Diabetes mellitus: Data adjusted with age, sex, smoking and drinking habits, chronic kidney disease, and other diseases (hypertension, dyslipidemia, and hyperuricemia). Dyslipidemia: Data adjusted with age, sex, smoking and drinking habits, chronic kidney disease, and other diseases (hypertension, diabetes mellitus, and hyperuricemia). Hyperuricemia: Data adjusted with age, sex, smoking and drinking habits, chronic kidney disease, and other diseases (hypertension, diabetes mellitus, and dyslipidemia).

## 4. Discussion

Our study showed that U.S. had 2.4-fold higher prevalence of hypertension, 2.6-fold higher prevalence of DM, and 1.7-fold higher prevalence of dyslipidemia, but almost same prevalence of hyperuricemia, compared to Japan. As anticipated, higher BMI was an independent risk factor for hypertension, DM, dyslipidemia, and hyperuricemia both in Japan and the U.S. after adjusting for age, sex, smoking and drinking habits, and other cardiovascular risk factors. Contrary to our hypothesis, the BMI cut-off above which the prevalence of these cardio-metabolic risk factors increased was significantly higher in the U.S. than in Japan. These results suggest there may be innate or cultural differences to both populations that define their risk profile with an increased BMI.

Our findings could be explained by the following hypotheses. Dietary habits, macronutrient content, and physical activity habits differ between Japan and U.S. For example, dietary fat intake and obesity levels significantly differ between the two countries [23]. U.S. diet derives approximately 33% of calories from fat [24] whereas fat intake represents approximately 26% of total calories in Japan [25]. Physical activity also differs in Japan compared to the U.S., as many in Japan use trains and buses and have been shown to have a higher level of physical activity [5]. Consistent with this, the prevalence of obesity is significantly higher in the U.S. than in Japan [23]. Of note, a previous study has shown that Asian university students were influenced by the U.S. culture after they came to the U.S., and they began to skip breakfast more often, eat more snacks with more salt and sugar, eat more fast food when they eat out, and consume more fats/sweet and dairy products, and less vegetables [26]. Similar findings were reported in a study of Chinese immigrant women who were found to have increased energy density of the diet, percent of energy from fat, and sugar intake after moving to the U.S. [27]. A previous NHANES study showed that the risks of obesity increases in foreign-born U.S. residents with time living in the U.S. [28]. The study also showed obesity prevalence was significantly higher in those born in the U.S. than those who had been in the U.S. for <1 year [28]. These data suggest that dietary and lifestyle habits in the U.S. are among the most important factors for developing obesity. Considering the higher rates of obesity in the U.S., it may be expected that higher BMI cut-off would associate with cardio-metabolic risk factors. Our study was not able to compare the physical activities between Japan and U.S. because of the difference of questionnaire about physical activities. However, we were able to check the calorie intake and the rate of the three major nutrients intake, and compared them between Japan and U.S. The calorie intake were similar between Japan and U.S. (2104 kcal vs. 2133 kcal), but Americans had more calories from fat than Japanese (33.5% vs. 16.3%, *p* < 0.001). In contrast, Americans had less calories from carbohydrate (50.8% vs. 64.2%, *p* < 0.001) and protein (15.7% vs. 19.5%, *p* < 0.001) than Japanese. Of note, we were not able to ascertain intake of sugar. Nevertheless, these results suggest that differences in the diet composition may contribute to the cardio-metabolic risk profiles differently in the U.S. versus Japan.

Alternatively, it is possible that other unidentified factors contribute to the increased risk of cardio-metabolic risk factors with lower BMI cut-offs in Japan than the U.S. such as underlying genetic predisposition. We are unaware of any data on genetic predisposition to metabolic syndrome in Japan or other Asian countries as compared to the U.S. It is important to note that WHO has identified a BMI of 23 kg/m^2^ or higher as an additional trigger points for public health action in Asian populations representing increased risk, where as a BMI of 27.5 kg/m^2^ or higher as representing high risk. WHO currently recommends that BMI of 18.5–23 kg/m^2^ is associated with acceptable risk based on the results of a meta-analysis involving results from nine countries in Asia and other published work [7]. While our findings are consistent with the lower BMI cut-off, our data indicate that a BMI of 22 kg/m^2^ or more (an even lower cut-off) associates with significantly higher odds of cardio-metabolic risk factors.

This study had several limitations. First, a limitation of the study is that the specific study periods were not identical between Japan (2004–2010) and U.S. (2001–2006), although the difference is only a few years. There is evidence that the prevalence of metabolic syndrome, hypertension, dyslipidemia, and DM are increasing in Japan as the culture becomes more westernized [29]. If anything, the fact that the Japanese study period is later would tend to increase the relative frequency of these conditions which would be expected to lessen the differences between the two populations. Thus, our finding of significant differences between the level of BMI and metabolic risk is unlikely to be affected by this limitation. Second, we were unable to evaluate physical activity, and further work is needed to better understand the contribution of diet composition to overweight/obesity in Japan. Third, this study assessed the prevalence of dyslipidemia between Japan and U.S., this was defined as taking lipid-lowering medication or as a low-density lipoprotein cholesterol level of ≥140 mg/dL, a high-density lipoprotein cholesterol level of <40 mg/dL, or a triglyceride level of ≥150 mg/dL. Lipid-lowering medicine, like statins and fibrates, affects all the values of LDL cholesterol, HDL cholesterol and triglyceride. Therefore, we were unable to assess the individual lipid profiles separately. Fourth, our data was from a single center and it might not be representative of the Japanese population. A benefit of the population studied was the large number of subjects, and we evaluated a representative data from the National Health and Nutrition Survey (Kokumin Kenkou Eiyou Chousa) in Japan [30] and compared their characteristics with our data from St. Luke’s International Hospital. The results showed similar characteristics between the two datasets including an equal proportion of men (49.1% vs. 49.0%), similar height (164.3 cm vs. 164.1 cm), BMI (22.4 kg/m^2^ vs. 23.2 kg/m^2^, <1 kg/m^2^ difference), and drinking habits (62.1% vs. 59.1%). Thus, while our data was collected from volunteers as opposed to the general population, it does appear to represent the Japanese population. Finally, this study could not assess cardiovascular morbidity and mortality prospectively because our study is a cross-sectional study. Future studies are needed to evaluate whether these BMI cut-offs are associated with increased risk of disease prospectively.

In conclusion, obesity and overweight are associated with higher prevalence of hypertension, DM, dyslipidemia, and hyperuricemia both in Japan and U.S. Japanese BMI cut-off is significantly lower than the American cut-off that associates with cardiovascular risk factors. These data suggest that the definition of overweight/obesity may differ based on country and that certain factors may render the Japanese population more sensitive to smaller increments in BMI.

## Figures and Tables

**Table 1:** Study subjects’ characteristics between Japan and the United States of America (U.S.).

	Japan	U.S.	*p*
Number of subjects	90,047	14,734	
Age (years old)	46.3 ± 12.0	45.7 ± 20.1	<0.001
Sex (male)	49.1%	50.0%	0.090
Height (cm)	164.3 ± 8.7	167.7 ± 10.2	<0.001
Weight (kg)	60.8 ± 12.4	79.5 ± 20.2	<0.001
Body mass index (kg/m^2^)	22.4 ± 3.3	28.2 ± 6.4	<0.001
Smoking	40.6%	49.1%	<0.001
Drinking habits	62.1%	67.8%	<0.001
Hypertension	15.5%	37.8%	<0.001
Diabetes mellitus	4.2%	10.8%	<0.001
Fasting blood glucose (mg/dL)	99.4 ± 15.6	97.1 ± 33.2	<0.001
HbA1c (%)	5.10 ± 0.59	5.54 ± 0.97	<0.001
Dyslipidemia	35.6%	60.8%	<0.001
Total cholesterol (mg/dL)	201.4 ± 34.4	199.2 ± 44.1	<0.003
Low-density lipoprotein cholesterol (mg/dL)	116.8 ± 30.7	132.8 ± 44.9	<0.002
High-density lipoprotein cholesterol (mg/dL)	62.4 ± 15.6	53.9 ± 16.1	<0.001
Triglyceride (mg/dL)	100.3 ± 81.9	143.8 ± 135.7	<0.000
Hyperuricemia	13.5%	12.8%	0.020
Serum uric acid (mg/dL)	5.29 ± 1.42	5.34 ± 1.44	<0.001
Chronic kidney disease	5.7%	7.5%	<0.001

**Table 2:** Body mass index as a risk for hypertension, diabetes mellitus, dyslipidemia, and hyperuricemia.

		Japan			U.S.		
**Hypertension**		**OR**	**95% CI**	***p***	**OR**	**95% CI**	***p***
Body mass index	per 1 kg/m^2^ increased	1.230	1.222–1.239	<0.001	1.068	1.060–1.077	<0.001
**Diabetes mellitus**							
Body mass index	per 1 kg/m^2^ increased	1.170	1.157–1.182	<0.001	1.086	1.076–1.096	<0.001
**Dyslipidemia**							
Body mass index	per 1 kg/m^2^ increased	1.223	1.217–1.230	<0.001	1.073	1.065–1.081	<0.001
**Hyperuricemia**							
Body mass index	per 1 kg/m^2^ increased	1.157	1.148–1.166	<0.001	1.089	1.078–1.100	<0.001

OR, odds ratio; 95% CI, 95% confidence interval. Hypertension: Data adjusted with age, sex, smoking and drinking habits, chronic kidney disease, and other diseases (diabetes mellitus, dyslipidemia, and hyperuricemia). Diabetes mellitus: Data adjusted with age, sex, smoking and drinking habits, chronic kidney disease, and other diseases (hypertension, dyslipidemia, and hyperuricemia). Dyslipidemia: Data adjusted with age, sex, smoking and drinking habits, chronic kidney disease, and other diseases (hypertension, diabetes mellitus, and hyperuricemia). Hyperuricemia: Data adjusted with age, sex, smoking and drinking habits, chronic kidney disease, and other diseases (hypertension, diabetes mellitus, and dyslipidemia).
